# Genome-wide analysis identifies critical DNA methylations within NTRKs genes in colorectal cancer

**DOI:** 10.1186/s12967-021-02740-6

**Published:** 2021-02-16

**Authors:** Zijian Chen, Zenghong Huang, Yanxin Luo, Qi Zou, Liangliang Bai, Guannan Tang, Xiaolin Wang, Guangwen Cao, Meijin Huang, Jun Xiang, Huichuan Yu

**Affiliations:** 1 Department of Gastrointestinal Surgery, The Sixth Affiliated Hospital, Sun Yat-sen University, Guangzhou, China; 2 Department of Colorectal Surgery, The Sixth Affiliated Hospital, Sun Yat-sen University, Guangzhou, China; 3 Guangdong Institute of Gastroenterology, Guangdong Provincial Key Laboratory of Colorectal and Pelvic Floor Disease, The Sixth Affiliated Hospital, Sun Yat-sen University, Guangzhou, China; 4 Department of Colorectal and Anal Surgery, The Sixth Affiliated Hospital, Sun Yat-sen University, Guangzhou, China; 5 Department of Epidemiology, Second Military Medical University, Shanghai, China

**Keywords:** *NTRKs*, *NTRK3*, Methylation, Colorectal cancer, Prognosis

## Abstract

**Background:**

Neurotrophic tropomyosin receptor kinases (*NTRKs*) are a gene family function as oncogene or tumor suppressor gene in distinct cancers. We aimed to investigate the methylation and expression profiles and prognostic value of *NTRKs* gene in colorectal cancer (CRC).

**Methods:**

An analysis of DNA methylation and expression profiles in CRC patients was performed to explore the critical methylations within NTRKs genes. The methylation marker was validated in a retrospectively collected cohort of 229 CRC patients and tested in other tumor types from TCGA. DNA methylation status was determined by quantitative methylation-specific PCR (QMSP).

**Results:**

The profiles in six CRC cohorts showed that *NTRKs* gene promoter was more frequently methylated in CRC compared to normal mucosa, which was associated with suppressed gene expression. We identified a specific methylated region within *NTRK3* promoter targeted by cg27034819 and cg11525479 that best predicted survival outcome in CRC. *NTRK3* promoter methylation showed independently predictive value for survival outcome in the validation cohort (*P* = 0.004, HR 2.688, 95% CI [1.355, 5.333]). Based on this, a nomogram predicting survival outcome was developed with a C-index of 0.705. Furthermore, the addition of *NTRK3* promoter methylation improved the performance of currently-used prognostic model (AIC: 516.49 vs 513.91; LR: 39.06 vs 43.64, *P* = 0.032). Finally, *NTRK3* promoter methylation also predicted survival in other tumors, including pancreatic cancer, glioblastoma and stomach adenocarcinoma.

**Conclusions:**

This study highlights the essential value of *NTRK3* methylation in prognostic evaluation and the potential to improve current prognostic models in CRC and other tumors.

## Introduction

Colorectal cancer (CRC) is the second leading cause of cancer death over the past years [1, 2]. Survival outcomes and optimal regimens vary in CRCs. At this point, some clinicopathological risk factors, such as TNM stage, tumor size, and tumor differentiation, have been used to stratify the risk of CRC death. Unfortunately, they fail to accurately distinguish patients with different outcomes [3], and several molecular biomarkers are being investigated and applied in current models to increase their prognostic values [4, 5]. CRC arises with the accumulation of gene mutations and epigenetic alterations [6, 7]. Among them, the aberrant methylation in gene promoters is prevalent across multiple cancers, which can lead to the inactivation of tumor suppressor genes [8]. Some of these aberrant methylations have been discovered and used to serve as prognostic biomarkers for CRC [9, 10].

Neurotropic tropomyosin receptor kinase (*NTRK*) gene family, including *NTRK1*, *NTRK2* and *NTRK3*, encodes tropomyosin receptor kinases (TRK), which can induce cell proliferation, differentiation, apoptosis, and survival of neurons through the PI3K, RAS/MAPK/ERK and phospholipase C-gamma signalling transduction pathways [11, 12]. The aberrations of *NTRKs* gene function were widely known to play an oncogenic role in multiple cancers. Among them, *NTRKs* gene fusion was the best-characterized aberration, which promotes tumorigenesis through the constitutive activation of downstream cell growth and proliferative pathways [12]. The first TRK inhibitor, larotrectinib, has been approved by FDA for the treatment of advanced solid tumors with *NTRKs* gene fusion [13].

Similar to gene fusion, the aberrant expression of *NTRKs* gene is a critical event in cancers. *NTRK1* promoted proliferation and metastasis of cancer cells and lead to poor prognosis in multiple cancers [14–18], while it suppressed cell proliferation in neuroblastoma [19]. *NTRK2* was shown to serve as an oncogene in multiple cancers [20–23], and its increased expression was associated with poor outcome [24, 25]. Based on this, inhibition of *NTRK2*-encoded TRKB was shown to induce antitumor effects and cellular apoptosis [26, 27]. Similar to *NTRK1*, *NTRK3* has been demonstrated to be an oncogene in breast cancer and gastric cancer [28, 29], but it acts as a tumor suppressor gene in CRC, neuroblastomas, and head and neck squamous cell carcinoma [11, 30, 31].

The expression of *NTRKs* gene can be modulated by promoter methylation. A hypermethylated *NTRKs* gene promoter is associated with suppressed expression in multiple cancers, such as CRC [11, 32], neuroblastoma [33], glioma [34], ovarian cancer [35] and prostate cancer [36]. Given the critical role of *NTRKs* gene in multiple cancers, we therefore aimed to perform a comprehensive analysis of *NTRKs* gene on their methylation signature, expression profile and prognostic value in CRC using the methylation profile we previously established and the published dataset, and identify the optimal CpG sites from *NTRKs* gene region as methylation biomarkers that can be applied in the current clinical models of CRC to improve their prognostic values.

## Materials and methods

### Study cohorts

In the discovery set, the clinical information and methylation profiles of CRC tissues and matched normal tissues we previously established in Fred Hutchinson Cancer Research Center cohort (FHCRC cohort, GSE48684 [32], n = 105, normal = 41, cancer = 64) using Illumina Infinium HumanMethylation450 BeadChip (450K microarray) were combined with datasets of TCGA-COAD&READ (The Cancer Genome Atlas-colon and rectum adenocarcinoma, n = 326, normal = 41, cancer = 285) cohort [37] and three Gene Expression Omnibus cohorts (GSE83889 [38], n = 136, normal = 35, cancer = 101; GSE39582 [39], n = 585, normal = 19, cancer = 566; GSE87211 [40], n = 363, normal = 160, cancer = 203) to investigate methylation and expression profiles of *NTRKs* gene, and identify the critical CpG methylations within *NTRKs* genes in CRC.

For the validation cohorts, we included 229 patients with histologically confirmed, stage I-IV CRC who underwent curative resection at the Sixth Affiliated Hospital of Sun Yat-sen University between 2009 and 2012. The patients were selected according to the exclusion criteria, including hereditary cancer, inflammatory bowel disease, and multiple primary cancers. To avoid a potential effect of chemotherapy on genomic methylation status, patients received chemotherapy before curative resection when tissue sample was collected were excluded. Patients were treated and followed according to the National Comprehensive Cancer Network guideline-based institutional protocol as previously described [41–43.]. Briefly, patients were followed at least every 3 months for the first 2 years and every 6 months for years three to five, in which cancer recurrence was screened by physical examination and cancer biomarkers, and a sequential computerized tomography scan with evidence of the disease followed by biopsy was applied to confirm the recurrence. To externally validate the findings in colorectal cancer and other cancers, we used the methylation array data and clinical information of 23 TCGA cohorts including a colon cancer cohort, a rectal cancer cohort and 21 cohorts of other tumor types, which was referred as external validation set.

**Fig. 1:**
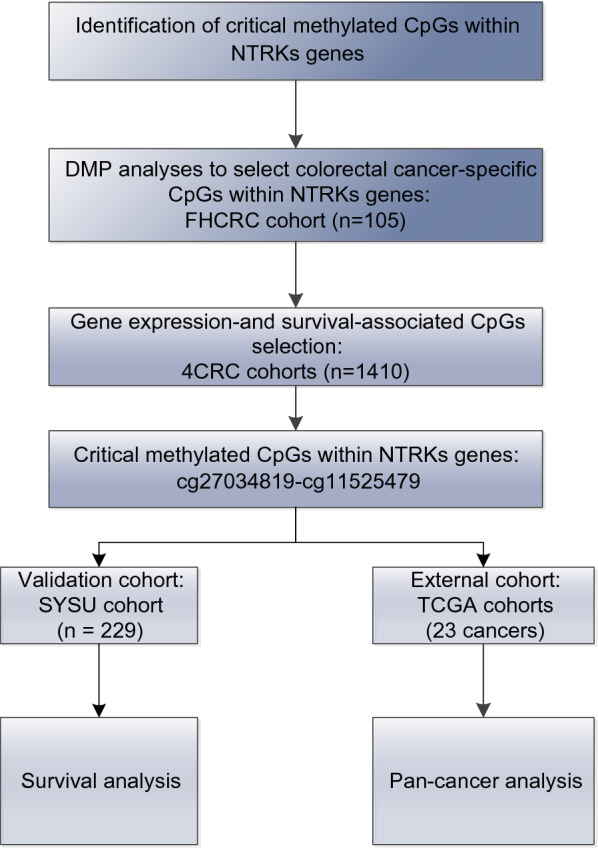
The flow diagram of cohort analyses. The Fred Hutchinson Cancer Research Center (FHCRC) cohort was an in-house retrospective cohort with 64 colorectal cancer (CRC) samples and 41 normal colon samples. The DNA methylation profile of FHCRC cohort was used to identify the differentially methylated probes (DMPs) within NTRKs genes between tumor and normal samples. We further used the clinical information, DNA methylation and mRNA expression profiles of TCGA-COAD & READ, GSE83889, GSE39582 and GSE87211 cohorts that prospectively enrolled patients with CRC to screen the critical methylated CpG sites within *NTRKs* genes. The SYSU (Sun Yat-sen University) cohort was an in-house retrospective cohort of 229 patients diagnosed with CRC that was used to validate the identified critical methylated CpGs. The external cohort is a set of 23 tumor types from TCGA, which was used to demonstrate the significance of identified critical methylated CpGs in pan-cancer

The cohort disposition for data analysis was illustrated in Fig. 1. The Institutional Review Board at the Sixth Affiliated Hospital of Sun Yat-sen University approved this study, and all the included patients have been given the written informed consent.

### Tissue collection

Formalin-fixed, paraffin-embedded (FFPE) CRC tissue specimens were available from the pathology registry. Hematoxylin and eosin (H&E) stained slides of cancer tissues were assessed by two experienced pathologists to confirm tumor histology based on the WHO classification of cancers and Fuhrman grade. Information of clinicopathological characteristics, such as age at diagnosis, gender, tumor size, tumor differentiation, and TNM stage, were extracted from the pathological reports and medical records.

### DNA isolation and methylation analysis

**Fig. 2:**
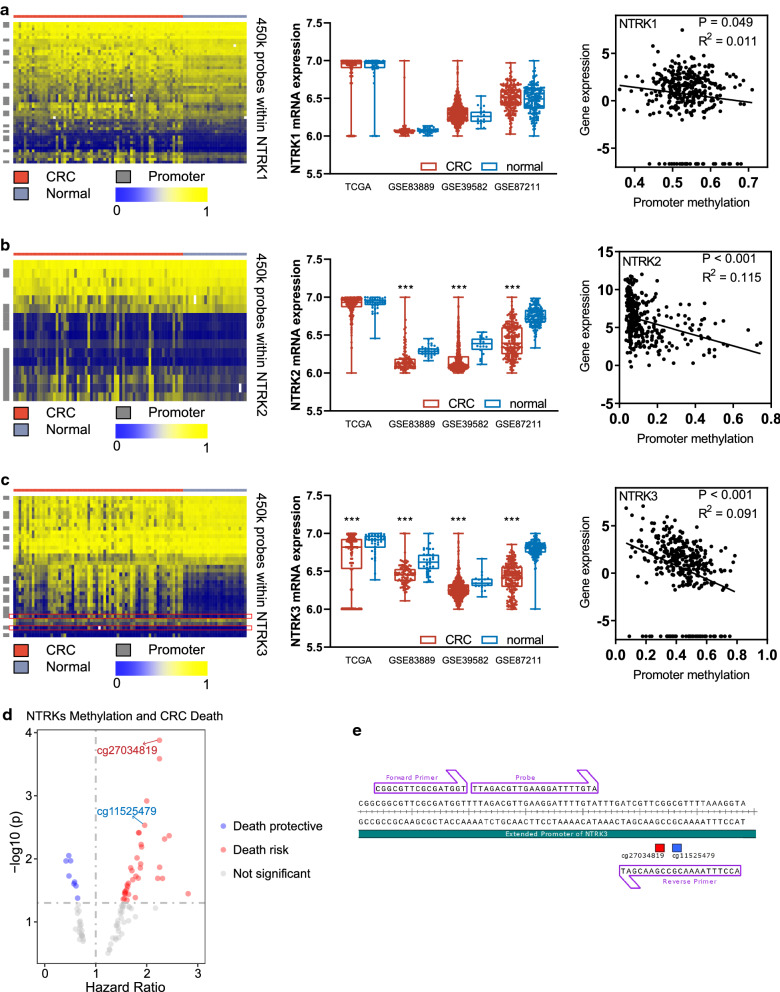
The methylation and expression profiles of *NTRKs* gene in CRC. **a**–**c**. The distribution and correlation of methylation and expression profiles of *NTRK1*, *NTRK2* and *NTRK3* gene in CRC and normal mucosa tissue. The heatmaps showed the results of probe-dimensional hierarchical clustering analysis of FHCRC cohort based on β values of all probes within *NTRK1* (**a**, left), *NTRK2* (**b**, left), and *NTRK3* (**c**, left). Each row represented a probe and each column represented a CRC or normal mucosa sample in the heatmap. Probes targeting promoter region were annotated on heatmap left. The red lines annotated in the heatmap for *NTRK3* indicated cg27034819 (top) and cg11525479 (bottom). *NTRKs* gene expression signature in each cohort showed suppressed mRNA expression of *NTRK2* (**b**, middle) and *NTRK3* (**c**, middle) in CRC tissues compared with normal mucosa tissue. Differences between CRC tissue and normal mucosa tissue were assessed with student t-test, and P values summarized with asterisks were shown in the upper space of the plot (***P < 0.001). The Spearman correlation analysis between mean promoter methylation of gene expression in each NTRK gene was shown in right panel. **d** Volcano plot of statistical significance against hazard ratio for DFS among all CpG sites within NTRKs gene targeted by 450K microarray probes. The top-ranked significantly probe was annotated according to the P values in the univariate Cox analyses. See Additional file 1 for the full results of univariate Cox analysis relevant to this panel. **e**. A QMSP assay was developed using primers and probe targeting cg27034819–cg11525479 region

Genomic DNA from FFPE tissue was isolated using the QIAamp DNA Mini Kit (Qiagen) and bisulfite-treated using EZ DNA Methylation Kit (Zymo) according to the manufacturer’s instructions. Gene methylation was analyzed by quantitative methylation-specific PCR (QMSP) that was performed using an ABI Prism 7700 detection system (Biosystems) as previously described [11, 44]. The oligo sequences of primers and probes were presented in Additional file 1: Table S1. The primers and probe designed to target cg27034819 and cg11525479 were illustrated in Fig. 2e. Alu-C4 was used as a reference locus for normalization for input DNA. Each reaction mix was consisted of 0.45 µM primers, 0.15 µM probe, 1.5 mM MgCl2, 200 pM dNTPs, and 1.5 U HotStart Taq. The thermocycler conditions were as follows: 95 °C for 10 min followed by 45 cycles of 30 s at 94 °C, 30 s at 60 °C. All samples were run in duplicate in at least two independent reactions. The methylation status of each sample was determined using the percentage of methylated reference (PMR) method [11]. The PMR ≥ 50 and < 50 were defined as hypermethylation and hypomethylation respectively in QMSP assay.

### Molecular characterization of CRC

The CpG Island Methylator Phenotype (CIMP) status and microsatellite instability (MSI) status of a subset of the colorectal neoplasms were assessed as described previously [45]. The assessment of *KRAS* and *BRAF* mutation was performed in the Molecular Diagnostic Laboratory of the Sixth Affiliated Hospital of Sun Yat-sen University, as previously described [46].

### Statistical analysis

All the statistical analyses were conducted using SPSS 20 or R 3.6.1. The *NTRKs* genes mRNA expression between CRC tissues and normal tissues were compared using student t-test. The Spearman correlation analysis was used to explore the relationship between promoter methylation and gene expression of *NTRKs* genes in TCGA cohort. The characteristics of CRC cases with and without *NTRK3* promoter hypermethylation were compared using the Wilcoxon rank-sum tests or Chi-square tests. Kaplan–Meier curves and Log-rank tests were used to evaluate the prognostic factors of disease-free survival (DFS) among candidate variables. DFS was defined as the time from curative resection until local recurrence, distant metastasis, or last follow-up. In addition, Cox proportional hazards analyses were used to obtain HRs and corresponding 95% confidence intervals (CI) for the association between *NTRK3* promoter hypermethylation and DFS. The variables that were considered clinically relevant or showed a significant difference (*P* < 0.05) in univariate Cox regression were entered into multivariate Cox proportional-hazards regression model using the backward stepwise selection method. Based on this model, nomograms subjected to internal validation set were generated for predicting 3-year and 5-year DFS outcomes, and the concordance index (C-index) was calculated to evaluate the predictive accuracy. The comparison between *NTRK3* hypermethylation and known prognostic factors was assessed using likelihood ratio (LR) and Akaike information criterion (AIC) in competing models including or not including *NTRK3* hypermethylation. In general, the model with a lower AIC and a higher LR is considered a better model. To validate these findings in colorectal and other cancers, the univariate Cox regression analysis of *NTRK3* hypermethylation on survival outcome was re-performed in 23 TCGA cohorts. In TCGA dataset, the normalized β value of cg27034819 and cg11525479 (adjacent to cg27034819), targeting the downstream region of the promoter region in *NTRK3*, was extracted from 450K microarray and its association with survival outcome was analyzed. A P value < 0.05 was considered statistically significant with a two-tailed test.

## Results

### *NTRKs* gene was commonly suppressed by DNA methylation in CRC

Using the methylation profile we previously generated through 450K microarray, we found *NTRKs* gene, including *NTRK1, NTRK2* and *NTRK3*, had more frequently methylated promoters in CRC samples when compared with matched normal mucosae (*NTRK1*, cancer = 0.444, normal = 0.397, *P* = 0.012; *NTRK2*, cancer = 0.251, normal = 0.167, *P* < 0.001; *NTRK3*, cancer = 0.395, normal = 0.144, *P* < 0.001; Fig. 2; Additional file 1: Table S2). In support of methylation analysis results, we found the mRNA expression of *NTRK2* and *NTRK3* in CRC samples was commonly lower than that in normal mucosae using the expression profiles in four CRC cohorts (n = 1410). However, no significant difference was observed in *NTRK1* mRNA expression (Fig. 2). Moreover, the expression of *NTRKs* gene is negatively correlated with their mean methylation of promoter (*NTRK1*, *P* = 0.049, R^2^ = 0.011; *NTRK2*, *P* < 0.001, R^2^ = 0.115; *NTRK3*, *P* < 0.001, R^2^ = 0.091; Fig. 2).

### A specific methylated region within *NTRK3* promoter best predicted CRC death

We next sought to identify the CpG site that could best predict CRC death and be feasibly used in a clinical assay. In the Cox proportional hazards analyses of 450K microarray probes targeting genomic loci within *NTRKs* gene, we found that the methylation of most CpG sites targeted by these probes was associated with poor survival outcomes in CRC (Fig. 2d, Additional file 1: Table S3). Among them, the methylation of cg27034819 was top-ranked for predicting CRC death. Of note, we found the probe cg11525479 was very close to cg27034819 in their targeting loci (Fig. 2e), and the methylation of cg11525479 also had a predictive value for CRC death that was superior to most probes. These results suggested that the specific region within *NTRK3* promoter targeted by cg27034819 and cg11525479 could be used to stratify the death risk of CRC. Interestingly, the methylation of this specific region was shown to be associated with the loss of NT3-dependent tumor suppressor gene function of *NTRK3* in our previous in vitro and in vivo study [11]. Therefore, a QMSP assay for determining the methylation of this specific region within *NTRK3* promoter was developed (Fig. 2e).

### Cohort validation of *NTRK3* methylation for prognostic significance

We further validated the predictive value of this candidate region within *NTRK3* promoter in our institutional cohort. The baseline characteristics of this validation cohort were summarized in Table 1. *NTRK3* promoter hypermethylation was observed in 26 of 229 patients (11.35%), and it was more frequent in patients with MSI (*P* = 0.015; Table 1). Moreover, *NTRK3* promoter hypermethylation was associated with *KRAS* mutation (*P* = 0.001; Table 1). For other characteristics relevant to clinical outcomes of CRC, they did not show significant difference between patients with *NTRK3* promoter hypermethylation and hypomethylation, including age, sex, tumor size, tumor differentiation, lymphovascular invasion, perineural invasion, TNM stage, CIMP, *BRAF* mutation, Ki-67, CA19-9, and CEA.

**Table 1:** Baseline characteristics and *NTRK3* promotor methylation status among all patients with CRC in validation cohort

Variables	Total	*NTRK3* promotor methylation		*P* value
		Hypomethylation	Hypermethylation	
Age (median = 62 years)				
< 62	111	100	11	
≥ 62	118	103	15	0.504
Sex				
Female	100	88	12	
Male	129	115	14	0.786
Tumor localization				
Colon	126	114	12	
Rectum	103	89	14	0.334
Tumor size (median = 4.5 cm)				
< 4.5	104	90	14	
≥ 4.5	123	111	12	0.382
Unknown	2			
Tumor differentiation				
High or moderate	191	168	23	
Poor	38	35	3	0.584
Lymphovascular invasion				
No	210	188	22	
Yes	17	13	4	0.219
Unknown	2			
Perineural invasion				
No	208	183	25	
Yes	19	18	1	0.611
Unknown	2			
TNM stage				
I–II	143	123	20	
III–IV	85	79	6	0.112
Unknown	1			
CIMP				
Negative	222	198	24	
Positive	7	5	2	0.145
Microsatellite status				
MSS	125	113	12	
MSI	51	39	12	0.015
Unknown	53			
*KRAS*				
Wild-type	119	110	9	
Mutation	62	46	16	0.001
Unknown	48			
*BRAF*				
Wild-type	174	151	23	
Mutation	8	6	2	0.674
Unknown	47			
Ki-67				
≤ 25%	100	89	11	
> 25%	84	72	12	0.502
Unknown	45			
CA19-9				
≤ 37	179	160	19	
> 37	32	29	3	0.833
Unknown	18			
CEA				
≤ 5	158	138	20	
> 5	57	54	3	0.122
Unknown	14			

CIMP: CpG island methylator phenotype; *KRAS*: kirsten rat sarcoma viral oncogene; *BRAF*: B-Raf proto-oncogene, serine/threonine kinase; Ki-67: kiel67 antigen; CA19-9: carbohydrate antigen 19-9; CEA: carcinoembryonic antigen

**Fig. 3:**
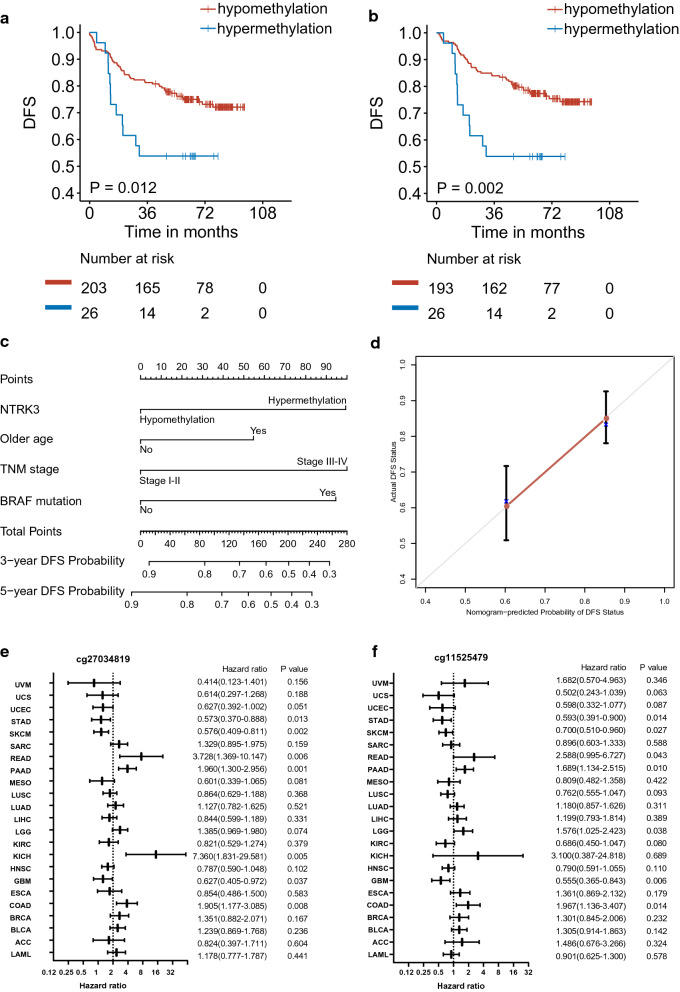
Prognostic significance of *NTRK3* promoter methylation. **a**, **b** Kaplan–Meier curves of disease-free survival according to *NTRK3* promoter methylation in CRC. Kaplan–Meier curves for the whole validation cohort (**a**) and stage I-III subgroup (**b**) were shown. The P value for each log-rank test was presented in the plots. Number at risk showed the quantity of CRC patients with *NTRK3* hyper- or hypo- methylation and among them the quantity of survivors or dead patients respectively. **c**, **d** A nomogram and calibration curve for predicting DFS in CRC. A nomogram to predict individual patient-level 3-year, and 5-year DFS based on clinicopathological risk factors and *NTRK3* methylation (**c**). Calibration plots for the validation sample of the above nomogram (**d**). Actual DFS statue measured via Kaplan–Meier analysis is shown on the Y-axis, and the nomogram-predicted probability of DFS statue is shown on the X-axis. The average nomogram-predicted probability of DFS was plotted against actually observed DFS estimated by Kaplan–Meier. 95% confidence intervals of the Kaplan–Meier estimates are indicated with vertical lines. Grayline indicates the reference line, showing where an ideal nomogram would lie. Instructions for users: Locate the status on each variable axis, and draw a straight line up to the Points axis to determine how many points toward risk the patient should receive from each variable. Sum the points and locate this number on the Total Points axis. Draw a straight line down from the total points to the 3-year or 5-year DFS Probability axis to ascertain the patient’s specific possibility of maintaining DFS until 3 or 5 years. **e**, **f** Pan-cancer analysis of the prognostic significance of *NTRK3* promoter methylation. The forest plots showed the values of the HR and CI for the prediction of the survival outcomes in univariate Cox analysis for the methylation of cg27034819 (**e**) and cg11525479 (**f**) in 23 TCGA cancer types. The x-axes presenting HRs were log2-scaled

In the Kaplan–Meier curve, significantly worse DFS outcomes were observed in patients with *NTRK3* promoter hypermethylation compared to those with *NTRK3* promoter hypomethylation (*P* = 0.012; Fig. 3a). The prognostic value of *NTRK3* promoter methylation status was further confirmed by univariate Cox proportional hazards (*P* = 0.014, HR 2.194, 95% CI [1.169, 4.117]; Table 2). Next, in the light of multivariate analysis, *NTRK3* promoter hypermethylation was still a prognostic factor adjusted by age, TNM stage, and *BRAF* mutation (*P* = 0.004, HR 2.688, 95% CI [1.355, 5.333]; Table 2).

**Table 2:** Cox proportional hazard analyses on DFS in patients with CRC

Variables	DFS in the patients with I–IV stage CRC				DFS in the patients with I–III stage CRC			
	Univariate		Multivariate		Univariate		Multivariate	
	*P* value	HR [95% CI]	*P* value	HR [95% CI]	*P* value	HR [95% CI]	*P* value	HR[95%CI]
*NTRK3* hypermethylation	0.014	2.194 (1.169, 4.117)	0.004	2.688 (1.355, 5.333)	0.004	2.565 (1.354, 4.857)	0.015	2.630 (1.206, 5.734)
Older age	0.011	1.952 (1.168, 3.263)	0.060	1.723 (0.977, 3.038)	0.002	2.401 (1.364, 4.227)	0.010	2.334 (1.217, 4.477)
Male (vs. female)	0.574	1.153 (0.702, 1.894)			0.484	1.207 (0.713, 2.043)		
Rectal tumor (vs. colon tumor)	0.479	1.192 (0.733, 1.939)			0.341	1.087 (0.915, 1.291)		
Tumor size ≥ 4.5	0.056	1.655 (0.988, 2.772)			0.081	1.624 (0.943, 2.799)		
Poor differentiation	0.581	1.193 (0.637, 2.233)			0.553	1.220 (0.633, 2.353)		
Lymphovascular invasion	0.052	2.087 (0.995, 4.378)			0.022	2.387 (1.131, 5.038)	0.003	3.287 (1.465, 7.373)
Perineural invasion	0.350	1.455 (0.663, 3.195)			0.158	1.770(0.801, 3.912)		
Advanced TNM stage	0.003	2.125 (1.300, 3.473)	< 0.001	2.704 (1.528, 4.790)	0.019	1.854 (1.107, 3.103)	0.039	1.978 (1.034, 3.784)
CIMP positive status	0.265	1.935 (0.607, 6.169)			0.186	2.191 (0.685, 7.010)		
MSI	0.316	1.350 (0.751, 2.427)			0.138	1.599(0.860, 2.973)		
*KRAS* mutation	0.113	1.566 (0.899, 2.726)			0.067	1.743 (0.962, 3.157)		
*BRAF* mutation	0.010	3.376 (1.338, 8.519)	0.049	2.563 (1.004, 6.551)	0.003	4.122 (1.621, 10.485)	0.595	1.443 (0.373, 5.591)
Ki-67 > 25%	0.458	0.806 (0.456, 1.425)			0.675	0.877 (0.476, 1.617)		
CA19-9 > 37	0.078	1.780 (0.938, 3.377)			0.191	1.589 (0.794, 3.179)		
CEA > 5	0.095	1.591 (0.923, 2.741)			0.048	1.769 (1.005, 3.111)	0.772	1.122 (0.514, 2.447)

*NTRK3*: neurotropic tropomyosin receptor kinase 3; CIMP: CpG island methylator phenotype; MSI: microsatellite instability; *KRAS*: kirsten rat sarcoma viral oncogene; *BRAF*: B-Raf proto-oncogene, serine/threonine kinase; Ki-67: kiel67 antigen; CA19-9: carbohydrate antigen 19-9; CEA: carcinoembryonic antigen

In sensitivity analyses, *NTRK3* promoter methylation was still independently associated with poor DFS outcome after the exclusion of patients with stage IV disease (*P* = 0.015, HR 2.630, 95% CI [1.206, 5.734]; Fig. 3b, Table 2), CIMP (*P* = 0.003, HR 2.806, 95% CI [1.432, 5.500]; Additional file 1: Table S4), MSI (*P* = 0.008, HR 3.483, 95% CI [1.391, 8.717]; Table Additional file 1: S5), or *BRAF* mutation (*P* = 0.025, HR 2.603, 95% CI [1.125, 6.022]; Additional file 1: Table S6) in multivariate Cox analysis.

### A nomogram for predicting DFS in CRC patients

A nomogram for predicting 3-year and 5-year DFS outcome was generated using the variables from the multivariate Cox model, including *NTRK3* methylation, age at diagnosis, TNM stage, and *BRAF* mutation (Fig. 3c). The calibration curves for the nomogram were shown, and the C-index of the nomogram for predicting DFS was 0.705 (Fig. 3d).

### *NTRK3* methylation adds values to current prognostic panels

The model 1 had a lower AIC and a higher LR compared with the model 2 (AIC: 597.73 vs 600.69; LR: 6.91 vs 5.95, *P* = 0.005; Table 3), indicating that *NTRK3* hypermethylation alone is better in predicting prognosis than rough TNM staging alone. In the comparison between model 2 and 3, after *NTRK3* hypermethylation was added to TNM stage, a lower AIC and a higher LR were observed (AIC: 600.69 vs 592.41; LR: 5.95 vs 16.23, *P* = 0.002; Table 3). These results suggest *NTRK3* hypermethylation could increase prognostic values of TNM staging.

**Table 3:** Model fit among seven models including or not including *NTRK3* methylation status

Models	N	AIC	LR	*P* value
Model 1	219	597.73	6.91	
Model 2	219	600.69	5.95	0.005^a^
Model 3	219	592.41	16.23	0.002^b^
Model 4	219	516.49	39.06	
Model 5	219	513.91	43.64	0.032^c^

N: patient counts in each model; AIC: Akaike information criterion value; LR: likelihood ratio

Model 1 includes *NTRK3* hypermethylation

Model 2 includes TNM stage (I, II, III)

Model 3 includes TNM stage (I, II, III), *NTRK3* hypermethylation

Model 4 includes age at diagnosis, sex (m/f), location(left colon, right colon, rectum), differentiation (well, moderate, poor), lymphovascular invasion, perineural invasion (n/y), T stage (T1, T2, T3, T4), N stage (N0, N1, N2), negative lymph node number, preoperation CEA, chemotherapy(n/y)

Model 5 includes *NTRK3* hypermethylation and all variables in Model 4

^a^*P* values for the LR test in model 1 compared with model 2; ^b^*P* values for the LR test in model 2 compared with model 3; ^c^*P* values for the LR test in model 4 compared with model 5

To determine the values of *NTRK3* hypermethylation in commonly-used models using multiple clinicopathological variables, model 4 was built using the variables included in the model recommended by AJCC [47, 48]. As expected, after *NTRK3* hypermethylation was included, model 5 had a lower AIC and a higher LR in comparison to model 4 (AIC: 516.49 vs 513.91; LR: 39.06 vs 43.64, *P* = 0.032; Table 3). Thus, the model recommended by AJCC may get increased discriminatory ability in predicting prognosis with *NTRK3* hypermethylation.

### Prognostic significance of *NTRK3* methylation in multiple tumors

Both the methylation of cg27034819 and cg11525479 were analyzed on their associations with survival outcome in 23 tumors using TCGA methylation profiles generated by 450K microarray. Overall, similar to the conflicting findings from in vitro and in vivo studies on *NTRKs* gene, the association of their methylations with survival outcome varied in different tumors. The hypermethylation of cg27034819 was significantly associated with worse survival outcome in colon adenocarcinoma (COAD; *P* = 0.008, HR 1.91, 95% CI [1.18, 3.09]), rectum adenocarcinoma (READ; *P* 0.006, HR 3.73, 95% CI [1.37, 10.15]), kidney chromophobe (KICH; *P* = 0.005, HR 7.36, 95% CI [1.83, 29.581], and pancreatic adenocarcinoma (PAAD; *P* = 0.001, HR 1.96, 95% CI [1.30, 2.96]) cohorts. However, it was significantly associated with better survival outcome in glioblastoma multiforme (GBM; *P* = 0.037, HR 0.63, 95% CI [0.41, 0.97], skin cutaneous melanoma (SKCM; *P* = 0.002, HR 0.58, 95% CI [0.41, 0.81]) and stomach adenocarcinoma (STAD; *P* = 0.013, HR 0.57, 95% CI [0.37, 0.89]) cohorts (Fig. 3e). In cg11525479 methylation analysis, a similar predictive value for worse survival were found in COAD, READ, and PAAD, and a similar predictive value for better survival were found in GBM, SKCM and STAD (Fig. 3f). These results suggested a robust prognostic value of the methylation of the specific promoter region targeted by cg27034819 and cg11525479 in multiple tumors.

## Discussions

In this study, we found the *NTRKs* gene promoter was more frequently methylated in CRC compared to normal mucosa, which was associated with suppressed expression of *NTRK2* and *NTRK3*. Through a screen of probes targeting *NTRKs* gene, we identified a specific methylated region within *NTRK3* promoter targeted by cg27034819 and cg11525479 that was the most promising prognostic marker for CRC. We developed a QMSP assay to determine the methylation of this region that could be easily applied in clinical assay and validate its predictive value for survival outcome in a cohort of 229 CRC patients and 23 TCGA cohorts including a colon cancer cohort, a rectal cancer cohort and 21 cohorts of other tumor types. Using *NTRK3* promoter methylation, age, TNM stage, and *BRAF* mutation, a novel nomogram predicting DFS outcome was developed and validated with a good prognostic performance. Also, we investigated the values of *NTRK3* promoter methylation that added to current prognostic panels, in which we observed a meaningful performance improvement of AJCC model and TNM staging alone after the introduction of *NTRK3* promoter methylation.

The conflicting findings in previous studies have revealed the complicated roles of *NTRKs* in different cancers. In our results, the mRNA expression of *NTRK2* and *NTRK3* was commonly lower in CRC samples in comparison to normal tissues, while the difference was not observed for *NTRK1*. In addition, this decreased expression of *NTRKs* was associated with promoter methylation. These results indicated that *NTRK2* and *NTRK3* may play a more important role of tumor suppressor in CRC, and methylation silencing of *NTRK2* and *NTRK3* would contribute more to CRC tumorigenesis. However, a decreased mRNA expression of *NTRK1* was found and attributed to the methylated promoter in neuroblastoma and ovarian cancer [33, 35].

In the discovery set, we identified the CpG site targeted by cg27034819 as the most promising methylation biomarker for prognosis, and further assessed its prognostic value in other cohorts. To apply this high-throughput screen finding to a clinical assay in large cohorts of patients, we developed a PCR-based assay covering this genomic region that can be easily used with low cost. The robustness of this assay includes the finding that the probe cg11525479 that was very close to cg27034819 also had a prognostic value superior to most probes. The use of this assay in our previous in vitro study on *NTRK3* also strengthen the reliability [11]. Using this assay in our CRC cohort, we found that *NTRK3* promoter hypermethylation was associated with worse DFS validation. This association maintained after the adjustment with all clinicopathological predictors. Furthermore, we revealed that *NTRK3* promoter hypermethylation is highly associated with MSI and *KRAS* mutation that is known as response biomarkers for cancer treatment and have conflicting predictive value for survival [4]. Therefore, we conducted sensitivity analyses excluding the patients with MSI or *KRAS* mutation. Consequently, the adverse impact of these molecular phenotypes on our methylation biomarker was minimal or not observed. Therefore, we are convinced that *NTRK3* promoter methylation determined by the QMSP assay we developed was an independent prognostic factor in CRC.

We used AIC and likelihood-ratio test to compare the discriminatory ability of predictive models for survival outcome as previously described [49]. First, *NTRK3* methylation status is better than rough TNM stage (I, II, III) in predicting prognosis in CRC. Then, the addition of *NTRK3* promoter methylation status in TNM stage and the AJCC models was shown to improve the predictive performance for DFS in CRC patients. Thus, *NTRK3* promoter methylation is a valuable prognostic marker in CRC patients.

In previous studies, *NTRK3* has been demonstrated to be an oncogene or a tumor suppressor gene in different cancer types [11, 28–31, 50, 51]. These conflicting findings from in vitro and in vivo studies is similar to the results of our cohort analyses on *NTRK3* methylation. In our analyses, *NTRK3* hypermethylation was associated with worse survival in some tumors, such as CRC, kidney chromophobe, and pancreatic adenocarcinoma, but it is related to a better outcome in other tumors, including glioblastoma multiforme, skin Cutaneous Melanoma, and stomach adenocarcinoma. *NTRK3* promoter methylation could serve as a prognostic marker in multiple cancers, and its prognosis role is different in various cancers.

We have to admit that our study has some limitations. First, despite the multiple supports from discovery, SYSU and TCGA cohorts, external validation for NTRK3 promoter methylation and the novel nomogram using the QMSP assay we developed would strengthen our findings. In addition, an independent validation in the cohort with methylation and transcriptomic profiles is essential for the association of *NTRKs* gene promoter methylation with its mRNA expression. Finally, although we excluded patients receiving chemo/radiotherapy before sample collection (neoadjuvant treatment) to avoid the confounding effect on genomic methylation and clonal selection, the retrospective nature of the SYSU cohort does not totally rule out other potential confounding factors.

## Conclusions

We found *NTRKs* gene was commonly suppressed by promoter methylation in CRC compared to normal mucosa. We identified the cg27034819–cg11525479 region within *NTRK3* promoter as the most promising predictive marker for survival outcome, and it was validated in our CRC cohort and 23 TCGA cohorts including a colon cancer cohort, a rectal cancer cohort and 21 cohorts of other tumor types. A novel nomogram included *NTRK3* promoter methylation and other independent predictors was developed. In addition, we observed a performance improvement of currently used prognostic models after the introduction of *NTRK3* promoter methylation. These findings have essential implications for prognosis stratification in clinical decision-making for CRC management.

## Supplementary Information

**Additional file 1. Table S1.** Primer and Probe Sequences. **Table S2.** The mean methylation of NTRKs promoter in CRC samples and matched normal mucosae in FHCRC cohort. **Table S3.** Univariate Cox analysis of probes targeting NTRKs gene in TCGA-COADREAD cohort. **Table S4.** Sensitivity analysis excluding patients with CIMP-positive status. **Table S5.** Sensitivity analysis excluding patients with MSI. **Table S6.** Sensitivity analysis excluding patients with BRAF mutation. **Table S7**. Sensitivity analysis excluding patients with KRAS mutation.

## Data Availability

The datasets used during the current study are available from the corresponding author on reasonable request.

## Abbreviations

ACC: Adrenocortical carcinoma
AIC: Akaike information criterion
BLCA: Bladder urothelial carcinoma
*BRAF*: B-Raf proto-oncogene, serine/threonine kinase
BRCA: Breast invasive carcinoma
CA 19-9: Carbohydrate antigen 19-9
CEA: Carcinoembryonic antigen
CI: Confidence intervals
CIMP: CpG island methylator phenotype
C-index: Concordance index
COAD: Colon adenocarcinoma
COADREAD: Colorectal adenocarcinoma
CRC: Colorectal cancer
DFS: Disease-free survival
ESCA: Esophageal carcinoma
FFPE: Formalin-fixed paraffin-embedded
FHCRC: Fred Hutchinson Cancer Research Center
GBM: Glioblastoma multiforme
HNSC: Head and neck squamous cell carcinoma
HR: Hazard ratio
Ki-67: Kiel67 antigen
KICH: Kidney chromophobe
KIRC: Kidney renal clear cell carcinoma
*KRAS*: Kirsten rat sarcoma viral oncogene
LAML: Acute myeloid leukemia
LGG: Brain lower grade glioma
LIHC: Liver hepatocellular carcinoma
LR: Likelihood ratio
LUAD: Lung adenocarcinoma
LUSC: Lung squamous cell carcinoma
MESO: Mesothelioma
MSI: Microsatellite instability
*NTRK1*: Neurotropic tropomyosin receptor kinase 1
*NTRK2*: Neurotropic tropomyosin receptor kinase 2
*NTRK3*: Neurotropic tropomyosin receptor kinase 3
*NTRKs*: Neurotropic tropomyosin receptor kinases
PAAD: Pancreatic adenocarcinoma
PMR: Percentage of methylated reference
QMSP: Quantitative methylation-specific PCR
READ: Rectum adenocarcinoma
SARC: Sarcoma
SKCM: Skin cutaneous melanoma
STAD: Stomach adenocarcinoma
SYSU: Sun Yat-sen University
TCGA: The Cancer Genome Atlas
TRK: Tropomyosin receptor kinases
UCEC: Uterine corpus endometrial carcinoma
UCS: Uterine carcinosarcoma
UVM: Uveal melanoma
