# Supplementary material for: Genome-wide analysis identifies critical DNA methylations within NTRKs genes in colorectal cancer
Source: J Transl Med. 2021 Feb 16;19:73. doi: 10.1186/s12967-021-02740-6 (PMC7885252; doi:10.1186/s12967-021-02740-6)
Supplement: Supplementary file 1 — Additional file 1. Table S1. Primer and Probe Sequences. Table S2. The mean methylation of NTRKs promoter in CRC samples and matched normal mucosae in FHCRC cohort. Table S3. Univariate Cox analysis of probes targeting NTRKs gene in TCGA-COADREAD cohort. Table S4. Sensitivity analysis excluding patients with CIMP-positive status. Table S5. Sensitivity analysis excluding patients with MSI. Table S6. Sensitivity analysis excluding patients with BRAF mutation. Table S7. Sensitivity analysis excluding patients with KRAS mutation. [file 12967_2021_2740_MOESM1_ESM.docx]

**Table S1 Primer and Probe Sequences**

| ***NTRK3* MethyLight Primer and Probe** | |
| --- | --- |
| Forward | CGGCGTTCGCGATGGT |
| Reverse | ACCTTTAAAACGCCGAACGAT |
| Probe | 6FAM-TTAGACGTTGAAGGATTTTGTA-MGBNFQ |

| ***Alu-C4* MethyLight Primer and Probe** | |
| --- | --- |
| Forward | GGTTAGGTATAGTGGTTTATATTTGTAATTTTAGTA |
| Reverse | ATTAACTAAACTAATCTTAAACTCCTAACCTCA |
| Probe | VIC-CCTACCTTAACCTCCC-MGBNFQ |

**Table S2 The mean methylation of *NTRKs* promoter in CRC samples and normal mucosae in FHCRC cohort**

| ***NTRKs*** | **The mean methylation of *NTRKs* promoter** | | ***P* value** |
| --- | --- | --- | --- |
|  | **cancer** | **normal** |  |
| ***NTRK1*** | 0.444 | 0.397 | 0.012 |
| ***NTRK2*** | 0.251 | 0.167 | < 0.001 |
| ***NTRK3*** | 0.395 | 0.144 | < 0.001 |

**Table S3 Univariate Cox analysis of probes targeting NTRKs gene in TCGA-COADREAD cohort**

| Probe | Gene | Relation to CpGI | *P* value.adjusted | Hazard.adjusted |
| --- | --- | --- | --- | --- |
| cg27034819 | *NTRK3* | Island | 0.0001 | 2.2500 |
| cg05901579 | *NTRK3* | Island | 0.0003 | 2.2500 |
| cg21956337 | *NTRK3* | Island | 0.0012 | 2.0000 |
| cg11525479 | *NTRK3* | Island | 0.0029 | 1.9600 |
| cg00008446 | *NTRK1* | Island | 0.0039 | 1.8500 |
| cg24845595 | *NTRK1* | N_Shore | 0.0039 | 1.8400 |
| cg03055346 | *NTRK1* | Island | 0.0043 | 2.4400 |
| cg26575633 | *NTRK1* | OpenSea | 0.0049 | 2.3500 |
| cg20664238 | *NTRK3* | N_Shore | 0.0059 | 1.8907 |
| cg14829066 | *NTRK3* | OpenSea | 0.0065 | 1.9000 |
| cg18534318 | *NTRK1* | Island | 0.0089 | 0.4720 |
| cg09936845 | *NTRK1* | OpenSea | 0.0097 | 1.8100 |
| cg01009697 | *NTRK2* | Island | 0.0108 | 0.5079 |
| cg02049180 | *NTRK1* | N_Shore | 0.0108 | 0.4178 |
| cg26687119 | *NTRK1* | N_Shore | 0.0121 | 1.8812 |
| cg19237923 | *NTRK1* | S_Shore | 0.0136 | 2.2532 |
| cg07252731 | *NTRK3* | Island | 0.0139 | 1.6856 |
| cg13504245 | *NTRK2* | N_Shore | 0.0139 | 1.8748 |
| cg26232187 | *NTRK3* | N_Shore | 0.0154 | 1.7106 |
| cg12039611 | *NTRK1* | S_Shelf | 0.0187 | 0.4754 |
| cg08921491 | *NTRK3* | OpenSea | 0.0189 | 1.7881 |
| cg08474334 | *NTRK1* | S_Shore | 0.0202 | 1.8334 |
| cg19084362 | *NTRK1* | N_Shelf | 0.0204 | 2.2163 |
| cg03888033 | *NTRK1* | Island | 0.0205 | 2.3098 |
| cg11286436 | *NTRK3* | N_Shore | 0.0214 | 1.7265 |
| cg03049917 | *NTRK3* | OpenSea | 0.0233 | 0.5854 |
| cg04920689 | *NTRK3* | Island | 0.0244 | 1.6416 |
| cg27314669 | *NTRK1* | S_Shelf | 0.0249 | 0.5727 |
| cg22402007 | *NTRK2* | N_Shore | 0.0259 | 1.6160 |
| cg18055585 | *NTRK3* | OpenSea | 0.0269 | 0.6134 |
| cg11108676 | *NTRK3* | Island | 0.0275 | 1.6268 |
| cg11793699 | *NTRK1* | Island | 0.0307 | 1.6056 |
| cg07418114 | *NTRK1* | OpenSea | 0.0328 | 1.5771 |
| cg04104526 | *NTRK1* | Island | 0.0336 | 1.5772 |
| cg09902251 | *NTRK3* | OpenSea | 0.0356 | 1.5654 |
| cg01357370 | *NTRK1* | Island | 0.0357 | 2.8127 |
| cg24867618 | *NTRK1* | OpenSea | 0.0360 | 1.5844 |
| cg03390245 | *NTRK1* | Island | 0.0366 | 1.6443 |
| cg26158180 | *NTRK3* | N_Shore | 0.0412 | 1.7764 |
| cg14447193 | *NTRK2* | OpenSea | 0.0420 | 0.6448 |
| cg11742207 | *NTRK3* | OpenSea | 0.0426 | 1.5625 |
| cg02619408 | *NTRK1* | N_Shore | 0.0435 | 1.5392 |
| cg08728702 | *NTRK1* | N_Shore | 0.0451 | 1.6133 |
| cg00626119 | *NTRK1* | S_Shore | 0.0522 | 1.5401 |
| cg25699759 | *NTRK3* | OpenSea | 0.0537 | 0.6586 |
| cg04627496 | *NTRK3* | Island | 0.0557 | 1.5084 |
| cg25306584 | *NTRK1* | OpenSea | 0.0565 | 1.5384 |
| cg09888330 | *NTRK1* | S_Shore | 0.0575 | 1.5223 |
| cg14384532 | *NTRK3* | Island | 0.0576 | 1.6462 |
| cg12499211 | *NTRK1* | S_Shelf | 0.0582 | 1.6628 |
| cg13965062 | *NTRK2* | Island | 0.0604 | 2.1721 |
| cg00577202 | *NTRK1* | S_Shore | 0.0606 | 1.5233 |
| cg10981464 | *NTRK1* | Island | 0.0615 | 1.6966 |
| cg00145961 | *NTRK3* | OpenSea | 0.0625 | 0.6318 |
| cg00242627 | *NTRK1* | Island | 0.0661 | 1.4829 |
| cg26328483 | *NTRK1* | OpenSea | 0.0670 | 0.6739 |
| cg11177059 | *NTRK1* | OpenSea | 0.0702 | 1.5118 |
| cg24707200 | *NTRK1* | S_Shelf | 0.0730 | 1.4850 |
| cg12710376 | *NTRK3* | OpenSea | 0.0731 | 0.6436 |
| cg01292475 | *NTRK2* | Island | 0.0828 | 1.7785 |
| cg16065473 | *NTRK3* | OpenSea | 0.0862 | 1.4988 |
| cg20041152 | *NTRK3* | N_Shelf | 0.0957 | 1.4337 |
| cg08470639 | *NTRK2* | Island | 0.0960 | 1.6450 |
| cg12995941 | *NTRK1* | Island | 0.0970 | 0.6956 |
| cg26428727 | *NTRK1* | Island | 0.1005 | 0.6792 |
| cg16438688 | *NTRK1* | Island | 0.1012 | 1.5543 |
| cg03610604 | *NTRK1* | OpenSea | 0.1058 | 1.4308 |
| cg03438943 | *NTRK1* | Island | 0.1110 | 0.7114 |
| cg10002133 | *NTRK3* | N_Shore | 0.1140 | 1.4135 |
| cg13620631 | *NTRK2* | OpenSea | 0.1143 | 1.4020 |
| cg09963892 | *NTRK3* | OpenSea | 0.1153 | 0.6925 |
| cg25827666 | *NTRK1* | S_Shore | 0.1252 | 1.4422 |
| cg11147094 | *NTRK3* | OpenSea | 0.1261 | 0.6924 |
| cg02039214 | *NTRK3* | OpenSea | 0.1300 | 0.6042 |
| cg18649438 | *NTRK1* | Island | 0.1368 | 0.7268 |
| cg03628748 | *NTRK2* | Island | 0.1399 | 1.4464 |
| cg15568960 | *NTRK3* | OpenSea | 0.1447 | 0.6753 |
| cg24251942 | *NTRK3* | OpenSea | 0.1513 | 0.7104 |
| cg13773705 | *NTRK3* | Island | 0.1550 | 1.5110 |
| cg25630380 | *NTRK1* | S_Shore | 0.1595 | 0.7226 |
| cg04704219 | *NTRK1* | S_Shore | 0.1664 | 0.7360 |
| cg14273545 | *NTRK2* | OpenSea | 0.1670 | 0.7381 |
| cg24438909 | *NTRK1* | N_Shore | 0.1670 | 0.7301 |
| cg13698224 | *NTRK2* | OpenSea | 0.1675 | 0.7190 |
| cg05012697 | *NTRK3* | N_Shore | 0.1775 | 1.4509 |
| cg00786657 | *NTRK3* | OpenSea | 0.1798 | 1.3342 |
| cg09926027 | *NTRK2* | Island | 0.1805 | 1.3910 |
| cg13654445 | *NTRK2* | OpenSea | 0.1892 | 0.7543 |
| cg20673279 | *NTRK1* | Island | 0.1896 | 0.6833 |
| cg10811945 | *NTRK1* | S_Shore | 0.1911 | 1.3940 |
| cg20839149 | *NTRK1* | S_Shelf | 0.2029 | 0.7519 |
| cg01441387 | *NTRK1* | OpenSea | 0.2243 | 1.2971 |
| cg13723118 | *NTRK2* | Island | 0.2700 | 1.2678 |
| cg04415780 | *NTRK1* | Island | 0.2783 | 1.2749 |
| cg09539438 | *NTRK2* | Island | 0.3129 | 1.2417 |

**Table S4 Sensitivity analysis excluding patients with CIMP-positive status**

| **Variables** | **Disease-free Survival** | | | | |
| --- | --- | --- | --- | --- | --- |
|  | **Univariate** | | **Multivariate** | | |
|  | ***P* value** | **HR[95%CI]** | | ***P* value** | **HR[95%CI]** |
| ***NTRK3* hypermethylation** | 0.016 | 2.229(1.158, 4.293) | | 0.003 | 2.806(1.432, 5.500) |
| **Older age** | 0.018 | 1.870(1.111, 3.147) | | 0.029 | 1.793(1.063, 3.025) |
| **Male (vs. female)** | 0.582 | 1.154(0.692, 1.924) | |  |  |
| **Rectal tumor (vs. colon tumor)** | 0.283 | 1.314(0.798, 2.162) | |  |  |
| **Tumor size ≥ 4.5** | 0.088 | 1.576(0.934, 2.658) | |  |  |
| **Poor differentiation** | 0.385 | 1.296(0.722, 2.325) | |  |  |
| **Lymphovascular invasion** | 0.188 | 1.760(0.758, 4.088) | |  |  |
| **Perineural invasion** | 0.314 | 1.500(0.681, 3.302) | |  |  |
| **Advanced TNM stage** | 0.002 | 2.244(1.356, 3.713) | | <0.001 | 2.497(1.493, 4.179) |
| **MSI** | 0.596 | 1.185(0.633, 2.218) | |  |  |
| ***KRAS* mutation** | 0.071 | 1.689(0.957, 2.982) | |  |  |
| ***BRAF* mutation** | 0.075 | 3.643(0.879, 15.095) | |  |  |
| **Ki-67 > 25%** | 0.289 | 0.723(0.397, 1.316) | |  |  |
| **CA19-9 > 37** | 0.070 | 1.852(0.951, 3.605) | |  |  |
| **CEA > 5** | 0.061 | 1.708(0.977, 2.987) | |  |  |

**Table S5 Sensitivity analysis excluding patients with MSI**

| **Variables** | **Disease-free Survival** | | | | |
| --- | --- | --- | --- | --- | --- |
|  | **Univariate** | | **Multivariate** | | |
|  | ***P* value** | **HR[95%CI]** | | ***P* value** | **HR[95%CI]** |
| ***NTRK3* hypermethylation** | 0.027 | 2.729(1.120, 6.653) | | 0.008 | 3.483(1.391, 8.717) |
| **Older age** | 0.011 | 2.575(1.248, 5.316) | | 0.032 | 2.237(1.071, 4.676) |
| **Male (vs. female)** | 0.150 | 1.672(0.831, 3.367) | |  |  |
| **Rectal tumor (vs. colon tumor)** | 0.741 | 0.889(0.442, 1.787) | |  |  |
| **Tumor size ≥ 4.5** | 0.124 | 1.746(0.859, 3.552) | |  |  |
| **Poor differentiation** | 0.144 | 1.837(0.812, 4.153) | |  |  |
| **Lymphovascular invasion** | 0.142 | 2.192(0.769, 6.245) | |  |  |
| **Perineural invasion** | 0.967 | 1.031(0.246, 4.329) | |  |  |
| **Advanced TNM stage** | 0.015 | 2.370(1.183, 4.750) | | 0.140 | 2.432(1.193, 4.957) |
| **CIMP positive status** | 0.685 | 0.049(0, 107099.072) | |  |  |
| ***KRAS* mutation** | 0.324 | 1.439(0.699, 2.963) | |  |  |
| ***BRAF* mutation** | 0.794 | 1.304(0.178, 9.576) | |  |  |
| **Ki-67 > 25%** | 0.551 | 0.809(0.402, 1.626) | |  |  |
| **CA19-9 > 37** | 0.125 | 2.132(0.810, 5.616) | |  |  |
| **CEA > 5** | 0.745 | 1.152(0.490, 2.712) | |  |  |

**Table S6 Sensitivity analysis excluding patients with BRAF mutation**

| **Variables** | **Disease-free Survival** | | | | |
| --- | --- | --- | --- | --- | --- |
|  | **Univariate** | | **Multivariate** | | |
|  | ***P* value** | **HR[95%CI]** | | ***P* value** | **HR[95%CI]** |
| ***NTRK3* hypermethylation** | 0.008 | 2.532(1.281, 5.006) | | 0.025 | 2.603(1.125, 6.022) |
| **Older age** | 0.079 | 1.687(0.942, 3.022) | |  |  |
| **Male (vs. female)** | 0.573 | 1.183(0.660, 2.118) | |  |  |
| **Rectal tumor (vs. colon tumor)** | 0.734 | 1.137(0.543, 2.382) | |  |  |
| **Tumor size ≥ 4.5** | 0.113 | 1.618(0.892, 2.934) | |  |  |
| **Poor differentiation** | 0.266 | 1.466(0.747, 2.874) | |  |  |
| **Lymphovascular invasion** | 0.021 | 2.974(1.175, 7.531) | | 0.019 | 3.209(1.208, 8.528) |
| **Perineural invasion** | 0.979 | 1.014(0.363, 2.834) | |  |  |
| **Advanced TNM stage** | 0.002 | 2.449(1.370, 4.375) | | 0.007 | 2.524(1.288, 4.948) |
| **CIMP positive status** | 0.717 | 0.049(0, 569721.990) | |  |  |
| **MSI** | 0.680 | 1.147(0.597, 2.206) | |  |  |
| ***KRAS* mutation** | 0.043 | 1.817(1.018, 3.242) | | 0.019 | 2.648(1.171, 5.988) |
| **Ki-67 > 25%** | 0.343 | 0.740(0.397, 1.379) | |  |  |
| **CA19-9 > 37** | 0.038 | 2.269(1.045, 4.929) | | 0.113 | 1.744(0.877, 3.470) |
| **CEA > 5** | 0.981 | 1.009(0.481, 2.114) | |  |  |

**Table S7 Sensitivity analysis excluding patients with KRAS mutation**

| **Variables** | **Disease-free Survival** | | | | |
| --- | --- | --- | --- | --- | --- |
|  | **Univariate** | | **Multivariate** | | |
|  | ***P* value** | **HR[95%CI]** | | ***P* value** | **HR[95%CI]** |
| ***NTRK3* hypermethylation** | 0.122 | 2.306(0.800, 6.651) | |  |  |
| **Older age** | 0.131 | 1.785(0.842, 3.784) | |  |  |
| **Male (vs. female)** | 0.631 | 1.202(0.568, 2.546) | |  |  |
| **Rectal tumor (vs. colon tumor)** | 0.734 | 1.137(0.543, 2.382) | |  |  |
| **Tumor size ≥ 4.5** | 0.158 | 1.736(0.807, 3.735) | |  |  |
| **Poor differentiation** | 0.254 | 1.765(0.665, 4.684) | |  |  |
| **Lymphovascular invasion** | 0.004 | 3.734(1.513, 9.218) | |  |  |
| **Perineural invasion** | 0.257 | 1.847(0.639, 5.343) | |  |  |
| **Advanced TNM stage** | 0.002 | 3.413(1.553, 7.500) | |  |  |
| **CIMP positive status** | 0.027 | 2.729(1.120, 6.662) | |  |  |
| **MSI** | 0.632 | 1.219(0.542, 2.742) | |  |  |
| ***BRAF* mutation** | 0.004 | 4.190(1.586, 11.071) | |  |  |
| **Ki-67 > 25%** | 0.488 | 0.762(0.353, 1.643) | |  |  |
| **CA19-9 > 37** | 0.291 | 1.780(0.611, 5.188) | |  |  |
| **CEA > 5** | 0.694 | 0.822(0.308, 2.189) | |  |  |
